# Effects of Virtual Rehabilitation Training on Post-Stroke Executive and Praxis Skills and Depression Symptoms: A Quasi-Randomised Clinical Trial

**DOI:** 10.3390/diagnostics14171892

**Published:** 2024-08-28

**Authors:** Rosaria De Luca, Antonio Gangemi, Maria Grazia Maggio, Mirjam Bonanno, Andrea Calderone, Vincenza Maura Mazzurco Masi, Carmela Rifici, Irene Cappadona, Maria Pagano, Davide Cardile, Giulia Maria Giuffrida, Augusto Ielo, Angelo Quartarone, Rocco Salvatore Calabrò, Francesco Corallo

**Affiliations:** IRCCS Centro Neurolesi Bonino-Pulejo, S.S. 113 Via Palermo, C. da Casazza, 98124 Messina, Italy; rosaria.deluca@irccsme.it (R.D.L.);

**Keywords:** apraxia, stroke, cognitive deficits, depressive symptoms, virtual reality rehabilitation

## Abstract

Introduction: Apraxia is a neurological disorder that is common after a stroke and impairs the planning and execution of movements. In the rehabilitation field, virtual reality (VR) presents new opportunities and offers advantages to both rehabilitation teams and individuals with neurological conditions. Indeed, VR can stimulate and improve cognitive reserve and abilities, including executive function, and enhance the patient’s emotional status. Aim: The objective of this research is to determine the effectiveness of VR in improving praxis skills and behavioural functioning in individuals with severe stroke. Methods: A total of 20 stroke patients were enrolled from February 2022 to March 2023 and divided by the order of their recruitment into two groups: the experimental group (EG: *n* = 10) received training to improve their praxis skills using VR whereas the control one (CG: *n* = 10) received the same amount of standard training. All patients underwent an evaluation using a psychometric battery that consisted of the Hamilton Rating Scale for Depression (HRS-D), Mini-Mental State Examination (MMSE), Frontal Assessment Battery (FAB), Spinnler and Tognoni test, and De Renzi and Faglioni test. Valuations were performed before rehabilitation (T0) and after its completion (T1). Results: Both groups demonstrated significant improvements post-intervention. The EG showed a greater enhancement in their MMSE scores (*p* = 0.002), and reductions in both ideomotor and constructive apraxia (*p* = 0.002 for both), compared to the CG. The VR-based training also resulted in significant improvements in their depression symptoms (HRSD scores improved, *p* = 0.012 in EG vs. *p* = 0.021 in CG). Conclusions: This pilot study suggests that VR could help reduce cognitive, constructive apraxia and ideomotor apraxia symptoms caused by stroke injury.

## 1. Introduction

Apraxia is a neurological disorder that impairs the planning and execution of movements, making it difficult to perform both simple and complex tasks upon instruction or imitation, even if these skills had previously been learnt [1]. Diagnosis requires the exclusion of weakness, sensory dysfunction, comprehension deficits, and incoordination as potential causes [2]. Apraxia is a condition caused by various types of acquired brain injuries including stroke, dementia, and cancer. This disorder is frequently observed in 28–51% of patients who have suffered a stroke in the left hemisphere of the brain [3]. However, recent studies indicate that right-hemisphere lesions can also cause this disorder, although with a lower prevalence of between 8% and 20% [4]. Daily actions such as dressing, eating, and taking care of oneself require fine movements and sequential actions [5]. Executive functions, which include planning, scheduling, and other higher cognitive capacities, play a crucial role in motor execution [6]. Therefore, understanding their impact is essential for effective treatment. As a consequence, failure to perform these actions can lead to feelings of frustration and depression. These symptoms can create a negative cycle in which depression further aggravates motor and cognitive difficulties, making recovery and management more difficult [7]. More than thirty subtypes of apraxia have been identified, and are classified according to various criteria such as the type of activity impaired, nature of the gestures, mode of input, type of errors, and body segment affected [2]. This present study focuses on ideomotor and constructive apraxia. Ideomotor apraxia involves a disconnection between the understanding of a skill and the corresponding motor actions. Patients understand what they have to do and the meaning of the action, but are unable to coordinate its execution. Constructive apraxia manifests itself with difficulties in copying, drawing, or building. Patients show difficulty in organising and assembling the parts of an object according to a specific shape or sequence [2]. The main treatment for apraxia consists of targeted rehabilitation, including occupational, cognitive, speech and physical therapies [8]. It is essential to start rehabilitation as soon as possible after the diagnosis of apraxia, especially in cases following acute injuries, in order to achieve the best results [9]. Rehabilitation for apraxia aims to enhance three areas. The first area concerns transitive gestures, in which the patient learns to use an object, recognises the associated action, and imitates the gesture even without the actual object. The second area focuses on intransitive symbolic gestures, such as hand signals (e.g., “hello”), and teaching the patient to perform these gestures in different contexts. The third area deals with non-symbolic intransitive gestures, which involve the imitation of meaningless gestures previously shown by the examiner. This approach allows the different dimensions of apraxia to be addressed, improving both motor skills and understanding of actions [10]. For constructive apraxia, treatment focuses on exercises aimed at improving the ability to organise and assemble parts of an object according to a specific shape or sequence. These exercises aim to enhance spatial and motor–cognitive planning skills [11]. Training sessions can be held either one-on-one or in groups, and can be led by therapists or nursing personnel. In these sessions, the emphasis is placed on developing underdeveloped skills, with the duration tailored to each individual’s ability to endure and the challenges they face in the activity. This approach takes into account a diverse array of strengths and weaknesses [10]. Despite their proven effectiveness, these methods have several limitations, including low repeatability and low patient involvement [12,13]. These limitations may compromise the consistency and effectiveness of the rehabilitation process, necessitating the exploration of innovative solutions [14]. It is therefore crucial to implement advanced technological approaches that not only address these limitations, but can also enhance rehabilitation outcomes [15]. Virtual reality (VR) is an innovative new methodology using computer technologies that creates two- or three-dimensional artificial environments, which closely resemble real-world settings, and allows users to interact with them in real time through hearing, sight, and touch [16]. VR tools such as the Virtual Reality Rehabilitation System (VRRS) allow daily activities to be simulated in a virtual environment, customising exercises according to the patient’s abilities [17]. In this way, training becomes more focused and interesting, increasing motivation and reducing boredom and frustration [18]. Thanks to virtual scenarios, the central nervous system receives a wide range of sensorial feedback, such as auditory, visual, and tactile, which seem to enhance synaptic plasticity and promote learning [16]. Several studies have indeed shown its effectiveness in improving both cognitive and motor skills [14,19,20]. For these reasons, this innovative technology can offer significant advantages in the treatment of patients with apraxia. 

The objective of this research is to determine the effectiveness of VR in improving praxis skills and behavioural functioning in individuals with a severe stroke.

## 2. Materials and Methods

### 2.1. Study Population

Twenty participants (mean ± SD of age: 48.30 ± 14.77 years; 50% male), all of whom had experienced a stroke, were recruited from the Neurorehabilitation Unit of the IRCCS Centro Neurolesi “Bonino-Pulejo” from February 2022 to March 2023 for this quasi-randomised study. They were assigned to either the experimental group (EG: *n* = 10) or the control group (CG: *n* = 10) based on the order of their recruitment. The sample size was determined by considering resource availability, feasibility within our clinical environment, and preliminary statistical power calculations that were aimed at detecting medium to large effects, which are suited for an exploratory study. A comprehensive description of both groups can be found in Table 1. Stroke patients and/or their caregivers were thoroughly informed about the study and provided their cooperation and written informed consent. The research protocol adhered to the principles outlined in the Helsinki Declaration of Human Rights and received approval from the local Ethics Committee (IRCCS-ME-CE 08/21).

### 2.2. Procedures

Patients were recruited based on the following criteria: (i) a stroke diagnosis (either haemorrhagic or ischemic) occurring at least 6 months prior; (ii) evidence of moderate to severe cognitive impairment (i.e., MMSE score of 12 or higher); (iii) age between 18 and 70 years; (iv) cognitive impairment confirmed through neuroradiological and clinical evaluations; (v) demonstrated good adherence to treatment; (vi) no history of psychiatric disorders (such as delirium or psychosis); and (vii) presence of constructive and/or ideomotor apraxia (assessed by the Spinnler and Tognoni and the De Renzi and Faglioni tests). 

Patients were excluded for the following reasons: (i) diagnosis of active epilepsy, (ii) significant sensory impairment (including loss of vision or hearing), (iii) serious medical conditions (such as heart or lung failure), (iv) substantial cognitive or behavioural difficulties that might disrupt participation in training, (v) inability to understand simple verbal instructions, as indicated by a Token test score below 4, and (vi) presence of debilitating behavioural changes or severe psychiatric symptoms. 

Psychometric evaluations consist of targeted neuropsychological assessments to evaluate cognitive abilities, praxis abilities, and depressive symptoms. Due to the different variables measured, standardised tests were selected for rapid administration to facilitate patient compliance. All participants in this study were given treatments aimed at rehabilitating praxis skills, along with standard psychological and physical therapies. The experimental group, consisting of 10 patients, underwent training to improve praxis skills using a virtual reality rehabilitation system (VRRS). The control group, on the other hand, which also consisted of 10 patients, participated in the same amount of standard training, but without the incorporation of a VR tool. Both treatments consisted of 24 sessions, with a duration of 60 min each, and were conducted three times a week for a period of eight weeks.

### 2.3. Psychometric Measures

Cognitive functioning was examined through the MMSE, a neuropsychological test that evaluates six domains: orientation in time and space, attention and computation, verbal registration, language, memory, and constructive application. Scores under 18 suggest substantial cognitive impairment, while scores ranging from 18 to 24 reflect moderate to mild impairment. A score of 25 is considered borderline, and scores between 26 and 30 signify normal cognitive functioning [21]. The presence or absence of depressive symptoms was assessed by the HRS-D scale, a clinical tool that includes 21 items, 4 of which are specifically intended to subtype depression. The score that ranges from 0 to 7 indicates the absence of depression, and a score of above 23 indicates a very severe depressive condition [22]. Executive functions were examined through the Frontal Assessment Battery (FAB). The FAB consists of six tasks that examine different aspects of executive functions: word generation, similarity, programming, mental flexibility, impulsivity control, self-criticism, and mindfulness. The score can vary between 0 and 18 and high scores reflect better executive functions [23].

The Spinnler and Tognoni test (1987) was used to assess constructive apraxia. The interviewer presents 7 sheets with figures of increasing difficulty that the subject must copy within a maximum time of 1 min. Each copy is given a score as follows: (i) 2 points if the spatial arrangement of the lines is correct; (ii) 1 point if the copy is partially defective, but not to the point of preventing identification of the model or at least parts of it; and (iii) 0 points if the reproduction is unrecognisable. The total score (range of 0–14) is the total of the partial results. Generally, the interpretation of the results suggests that as the score decreases, the impairment of praxis abilities increases [24]. 

Ideomotor apraxia was assessed by the De Renzi and Faglioni (1999) test. The test includes 24 gestures of which 12 are symbolic and 12 are non-symbolic. Each gesture can be reproduced a maximum of three times if the first attempt is not correct, and a score between 3 and 0 is awarded based on whether the reproduction is accurate on the first, second, or third attempt, or not at all (score range of 0–72). A diagnosis of apraxia is made with a score of less than 53, while a score between 53 and 62 indicates likelihood of disorder, and ideomotor apraxia is ruled out when the score is greater than 62 [25].

### 2.4. Praxis Abilities Exercise

Traditional treatment for post-stroke ideomotor and constructive apraxia relied on the use of tools such as paper and pencil and involved direct interaction between therapist and patient. The sessions aimed to improve intentional movements and coordination between thought and action. This approach also included psychological support, which was essential to motivate and support the patient. The method was individualised, adapting exercises according to the patient’s progress, to ensure effective recovery.

VR intervention focuses on the recovery of post-stroke ideomotor and constructive apraxia by stimulating the same cognitive areas as traditional methods through an innovative interface called Virtual Reality Rehabilitation System (VRRS). This system is organised in different rehabilitation packages: (i) motor module: includes exercises for body positioning, upper and lower body movements, mimicking expressions, and breathing; and (ii) cognitive module: includes exercises for logical–mathematical skills, executive functions, attention, and others. Within the cognitive module, there is a specific subdomain to stimulate skills related to ideomotor and constructive apraxia in stroke patients. The virtual rehabilitation program involves virtual exercises, such as puzzles and simulations of everyday actions, which are aimed at improving these skills through specific tasks. For a detailed description of the program and exercises performed, see Table 1 and Table 2.

### 2.5. Statistical Analysis 

Statistical analyses were conducted utilising Python 3.10.12 with the SciPy library (version 1.11.4). A significance level of *p* < 0.05 was utilised to evaluate statistical significance. Descriptive statistics, such as the mean, standard deviation, and range, were computed for each group to give a comprehensive summary of the characteristics of the dataset. The Chi-squared test was used to assess proportions, such as the distribution of categorical variables among groups. The Shapiro–Wilk test was utilised to evaluate normality of MMSE, FAB, HRS-D, Constructive Apraxia, and Ideomotor Apraxia scores distributions at T0 and T1. Due to the non-normal results and the study’s small sample size, non-parametric tests were chosen for further analyses. The Mann–Whitney U test was applied for inter-group comparisons, analysing differences between the EG and CG. The Wilcoxon signed-rank test was used for intra-group comparisons, assessing changes within each group from T0 to T1.

## 3. Results

**Figure 1:**
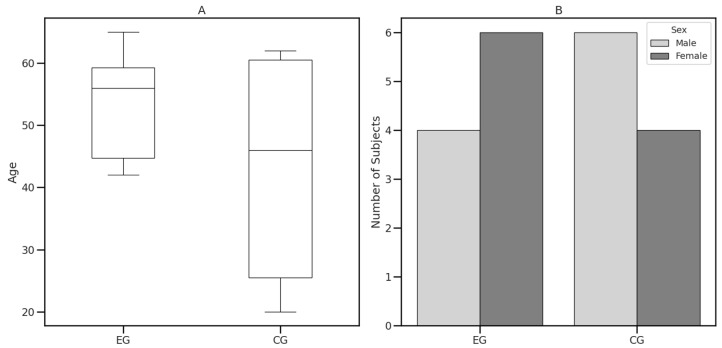
Demographic data. (**A**) Age distribution between groups. (**B**) Sex distribution between groups. EG = Experimental Group; CG = Control Group.

The experimental and control groups had similar age and sex distributions (Figure 1).

The descriptive statistics revealed comparable baseline characteristics between the two groups, with no significant differences in age (*p* = 0.29) and sex distributions (*p* = 0.65). However, there was a significant difference in education level (*p* < 0.001). For more details, see Table 3. 

Intra-group comparisons showed statistically significant changes across several measures within both the groups. Specifically, the MMSE scores significantly improved in both the EG and CG, with a 95% confidence interval (CI) from 2.13 to 4.67 (*p* = 0.002) for the EG, and a 95% CI from 0.91 to 2.29 (*p* = 0.002) for the CG. Similarly, the FAB scores in the EG showed significant improvement (95% CI from 0.96 to 1.63; *p* = 0.002) and less pronounced changes in the CG (95% CI from 0.31 to 1.36; *p* = 0.022). The HRSD scores also demonstrated significant improvements, with a 95% CI from −1.76 to −0.64 (*p* = 0.012) for the EG and a 95% CI from −2.20 to −0.40 (*p* = 0.021) for the CG. Both constructive apraxia and ideomotor apraxia also showed notable improvements, with a 95% CI from 2.47 to 4.13 (*p* = 0.002) and a 95% CI from 5.33 to 9.87 (*p* = 0.042) for the EG, and a 95% CI from −0.08 to 4.48 (*p* = 0.002) and a 95% CI from 1.08 to 11.12 (*p* = 0.009) for the CG, respectively. The EG exhibited a greater improvement compared to the CG in the MMSE, and with respect to constructive apraxia and ideomotor apraxia.

The Mann–Whitney test revealed significant differences between the EG and CG in the MMSE scores at both T0 and T1, with 95% CI from 2.13 to 4.67 (*p* = 0.002) and a 95% CI from 0.91 to 2.29 (*p* = 0.007) respectively, indicating a greater improvement in the EG. However, no significant differences were observed between the groups for the FAB scores at T0 (*p* = 0.306) and T1 (*p* = 0.568), HRSD scores at T0 (*p* = 0.761) and T1 (*p* = 0.704), constructive apraxia scores at T0 (*p* = 0.337) and T1 (*p* = 0.512), or ideomotor apraxia scores at T0 (*p* = 0.363) and T1 (*p* = 0.383).

**Figure 2:**
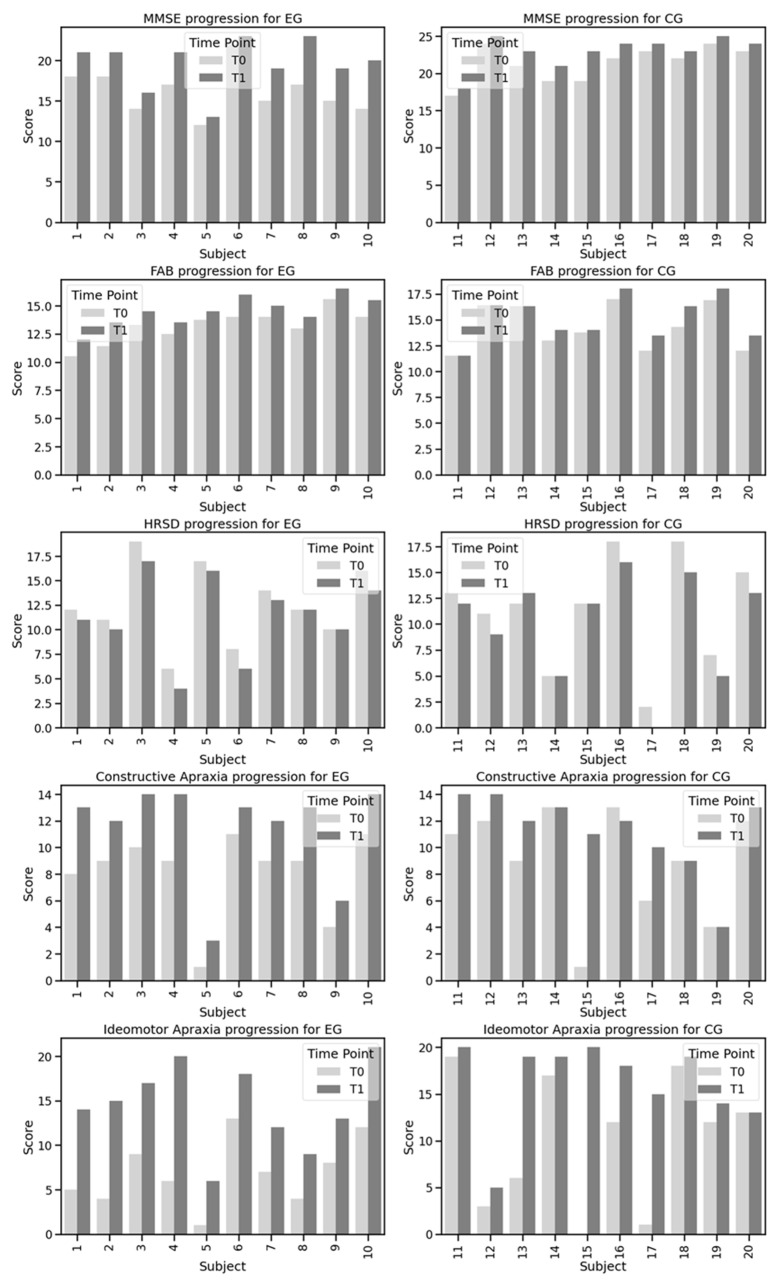
Scores progress over time. Experimental Group (EG); Control Group (CG); Mini Mental State Examination (MMSE); Frontal Assessment Battery (FAB); Hamilton Rating Scale for Depression (HRS-D).

**Figure 3:**
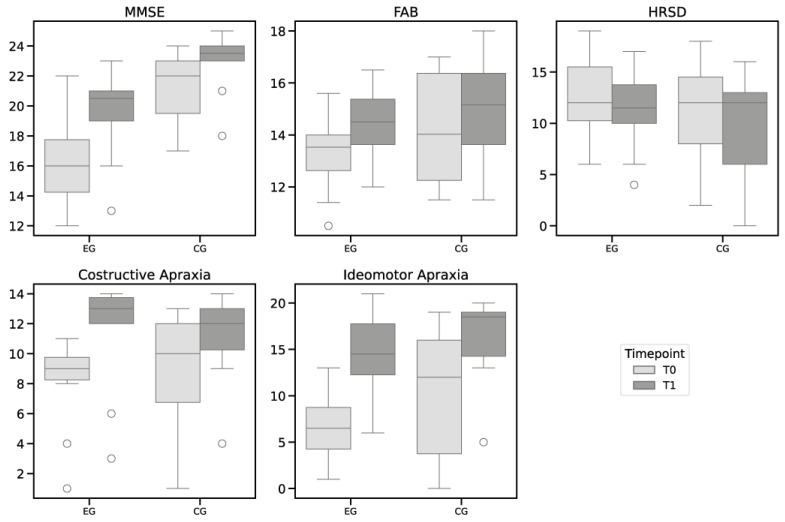
Scores pre- and post-Treatment. EG = Experimental Group; CG = Control Group.

The detailed results of the intra-group and inter-group analyses are reported in Table 4 and in Figure 2. The boxplots in Figure 3 illustrate the distribution of the scores at T0 and T1.

## 4. Discussion

This study examined the effectiveness of VRT on executive function and praxis skills and behavioural functioning in patients with a severe stroke. The results indicate that both the EG and CG showed significant improvements in several psychometric measures, including the MMSE, FAB, and HRSD. However, the experimental group, which received VRT treatment, had a greater improvement in global cognitive functioning than the CG.

The improvements in executive functions and depression are also greater in the EG than in the CG. This result is in agreement with other studies, which suggest that interventions for psychomotor functioning have knock-on effects in additional domains, such as executive functions and mood, according to a dynamic approach [26,27]. In a recent study, Shi et al. analysed the correlation between cognition and post-stroke depression and found that mood and executive functions were the core elements in the related networks, whereas psychomotor and attention functioning were the key bridging components in the networks [28]. Therefore, interventions designed to improve cross-network components, such as attention and psychomotor functioning, could also facilitate a more holistic post-stroke recovery of pivotal functions.

The results of this study are aligned with previous research highlighting the effectiveness of VRT in neuropsychological rehabilitation. Indeed, VRT could significantly improve cognitive and motor functions in stroke patients by offering an involving and interactional educational environment [29,30,31]. A meta-analysis by Laver et al. [32] noticed that the use of VRT is associated with significant improvements in the rehabilitation of motor and cognitive functions when compared to standard therapy.

Another important improvement observed in our group is related to praxis skills (constructive and ideomotor apraxia). This result is in line with our recent study [33] in which we observed that VR can also encourage both constructive and ideomotor praxis skills. Praxia consists of a cognitive component that is often neglected in the rehabilitation field, even though it significantly influences the daily life and personal relationships of the patients [34,35]. Praxia consists of planning, organisation, coordination, and motor and gestural synchronisation. The few studies on the topic have highlighted the potential effect of strategic training and gestural learning sessions on movement skills, cognitive functioning, and the outcomes with respect to the activities of everyday life in individuals with neurological conditions [36,37,38]. In particular, our study highlighted that the approach of using VR with audiovisual-augmented feedback could be effective for treating this skill. This could be due to the effect of VR training, which also acts on mirror neurons [33]. Recent studies [39,40] have shown that VR not only stimulates these neurons but also promotes neuroplasticity, making motor rehabilitation more effective and engaging for post-stroke patients than traditional methods. Mirror neurons, which are activated both when we perform an action and when we observe someone else perform it, are crucial in this process. During VRT, patients perform motor exercises while observing virtual avatars performing similar movements. This observation stimulates mirror neurons and helps in the reorganisation of the damaged brain areas, thanks to neuroplasticity [39]. As underlined by other studies [41,42], VR allows for performing a repetitive and task-oriented movement, with direct feedback on the movements performed (screen changes), which leads to an awareness of the movements implemented and the quality of the movements themselves. This VR feature leads to the patient feeling a sense of embodiment, which is generated by the movement of the arm and the consequent effects observed on the VR screen. This is possibly due to the influence of the VR intervention, which also acts on mirror neurons [33]. In addition to the involvement of mirror neurons [43], the cognitive and praxis improvements observed in our group could be due to the characteristics of VR, which allow for the combination of cognition, perceptions, and actions, along with the retrieval of stored movement patterns [33,43]. In fact, it has been shown that VR, through realistic and stimulating exercises, enables a better mastery of the different areas: sensory, motor, cognitive, and social [44]. Furthermore, recent studies have shown that VR can stimulate motor learning [45], as well as attentional, executive, and visuo-perceptual skills, through dual-task exercises [35]. The improvement in praxis ability might result from integrating cognitive exercises, imitation elements, and motor sequences. This approach promotes the execution of sequential movements and offers heightened visual and auditory stimulation, leading to a dual strengthening of both motor skills and cognitive processes [46]. Indeed, in a single case study by [47], who performed VR training on a 56-year-old man experiencing post-stroke ideomotor apraxia, this training resulted in a significant improvement of his apraxia symptoms following training in a virtual reality setting [20]. Finally, Rohrbach et al. carried out research involving stroke patients using a VR holographic simulation to enhance their use of pantomime tools. The findings indicated that the patients attained notably higher scores when using the VR-mediated holographic or dynamical cues [20]. This suggests that training with virtual reality allows for the transition to real objects, characterised by intrinsic perceptual (temperature, texture) and visuo-constructive (stereoscopic, deformability, interlocking) components, improving performance in daily activities [48]. Our findings, such as those observed in individuals with other neurological pathologies, e.g., multiple sclerosis, therefore, highlight the importance of treating apraxia using VR.

One of the main limitations of this study is the significant difference in the educational levels between the experimental and control groups. This may have influenced the results, as a higher level of education is often associated with a better cognitive reserve and better rehabilitation outcomes [49]. Without an adequate control for the educational level, it is difficult to determine whether the effects found are attributable to the intervention or simply to differences in educational background. To mitigate this problem in the future, it would be useful to adopt control measures, such as stratifying groups by educational level or including educational level as a control variable in statistical analyses. This approach could help isolate the effect of the intervention from the education-related influences. The small sample size is a significant limitation in this study, as it reduces the generalisability of the results to the entire population of patients with apraxia and compromises the ability to detect significant differences between the groups. This limitation may result from several factors, including the difficulty of recruiting sufficient participants who meet the inclusion criteria and the limited availability of patients to take part in the study. For these aspects, this study is to be understood as a pilot study whose results need to be confirmed with larger populations and long-term follow-up. To expand future research, it might be useful to create or adopt additional standardised tests that measure relevant variables that have not been previously explored, involve more participants and research centres in randomised trials, extend the observation period to assess the long-term effects of the variables under investigation, include neuroimaging, and collaborate with a larger team of researchers to enrich the study with diverse expertise.

The findings of this study carry significant clinical relevance. Integrating VRT into standard rehabilitation programs could significantly improve practice and cognitive abilities in patients with severe stroke. However, it is necessary to consider the training and resources needed to effectively implement VRT in rehabilitation settings.

## 5. Conclusions

In summary, this study gives preliminary evidence suggesting that VRT has potential effectiveness in improving praxis and cognitive abilities in patients with a severe stroke. Future research with larger samples and rigorous controls is necessary to validate these results and continue to explore the impact of VRT on further areas of neuropsychological and behavioural functioning.

## Figures and Tables

**Table 1:** Programs for people with ideomotor and constructive apraxia using either traditional and virtual therapy.

Domain	Objective of Therapy	Activities Carried Out through Traditional Therapy (Control Group)	Activities Carried Out through Therapy with VRRS (Experimental Group)
Ideomotor Apraxia	Therapy for ideomotor apraxia aims to improve the ability to perform intentional movements and coordination between thought and action in patients with this condition.	- Motor imitation exercises: imitate gestures and movements, guided by the therapist, to improve motor accuracy and coordination. - Motor sequencing activities: practice sequences of movements, such as dressing, to improve the ability to perform complex actions. - Gesture exercises: exercises to associate symbolic gestures with concrete meanings, improving the understanding and appropriate use of gestures.	- Daily action simulations: use everyday life scenarios, such as using money, to improve the ability to perform sequences of actions. - Virtual imitation exercises: imitate gestures shown in a virtual environment to develop accuracy and motor coordination. - Virtual life environments: explore virtual environments that simulate everyday life contexts to facilitate the practice of motor actions in realistic settings.
Constructive Apraxia	Therapy for constructive apraxia aims to improve drawing, construction, and object manipulation skills in patients with this condition.	- Manual construction exercises: consist of reproducing geometric drawings or objects using pencils or pens. - Drawing and copying exercises: involve copying drawings of objects to improve accuracy and reproduction of details. - Model building activities: use plasticine materials to build three-dimensional models following detailed instructions. - Cutting and glueing activities: work with scissors, paper, and glue to practice precise manipulation and hand control.	- Virtual construction simulations: consist of using VRRS to practice building and assembling digital objects, improving hand–eye coordination. - Virtual drawing and modelling exercises: allow the patient to create and modify virtual objects to develop accuracy and spatial representation. - Virtual puzzle exercises: involve the patient in assembling three-dimensional jigsaw puzzles to allow for improved visuomotor coordination, spatial understanding, and problem-solving skills, which are essential for meeting the challenges of constructive apraxia. - Virtual pathways: following virtual paths that require coordinated movement through complex virtual environments improves the ability to navigate and perform multiple tasks.

**Table 2:** Description of exercises used in traditional and virtual reality therapy sessions for ideomotor apraxia and constructive apraxia.

Domain	Individual Session Duration	VRRS Therapy			Traditional Therapy		
		Exercise	Description	Aim	Exercise	Description	Aim
Ideomotor apraxia	3 times a week for 60 min of traditional therapy or virtual reality therapy for 8 weeks	Virtual meal preparation	Simulation of food preparation, such as cutting and mixing.	Coordination and sequencing of complex movements.	Imitation of Movements	The patient imitates the hand and arm movements performed by the therapist.	Helps restore the ability to perform movements on command.
		Virtual money management	Count, manage, and give change in a virtual environment.	Strengthens computational and object manipulation skills.	Sequences of movements	The patient performs a series of movements in a specific sequence (e.g., bring a hand to the mouth, then raise an arm).	Improves the ability to perform sequences of motor actions.
		Farm	Simulation of virtual farming activities, such as cultivating and harvesting.	Improves motor sequence and coordination in complex tasks.	Gestures on command	The patient performs symbolic gestures such as waving, pointing, or mimicking the use of objects upon request.	Enhances the ability to perform learned gestures and actions on command.
		Virtual dressing	Clothing activities: buttoning shirts, putting on shoes.	Facilitates motor coordination and sequencing of complex movements.	Handling of objects	The patient manipulates everyday objects such as combs, pens or cups, performing appropriate actions with them.	Strengthens the ability to use objects functionally.
		Replica of gestures	Perform gestures or actions shown in VR simulations, such as greetings or orders.	Improves learning of symbolic gestures and imitative movements.	Acknowledgment of shares	The patient observes images of people performing actions and must recognise or describe the action.	Stimulates recognition and understanding of motor actions.
		Identify the action	Recognise and replicate specific actions shown in VR simulations.	Enhances the ability to understand and perform actions on command.	Dressing in Sequence	The patient performs the complete dressing sequence, from putting on socks and shoes to putting on a jacket and hat.	Strengthens the ability to perform complex, everyday motor sequences.
		Virtual supermarket	Simulate the purchase of products: take items from shelves, pay at checkout.	Enhances motor skills and management of daily actions.	Reordering of sequences	The patient must sequentially order pictures representing an action (e.g., washing hands).	Improves understanding of the logical sequence of motor actions.
Constructive apraxia	3 times a week for 60 min of traditional therapy or virtual reality therapy for 8 weeks	Virtual puzzle	Solve three-dimensional puzzles by fitting virtual pieces into a predefined pattern.	Strengthens spatial perception and hand-eye coordination.	Construction of geometric figures	The patient must construct simple geometric figures using blocks or puzzle pieces.	Improves ability to perceive and organise spatial shapes.
		Design and construction	Draw and build complex objects or structures in a virtual three-dimensional space.	Enhances spatial and motor planning skills.	Copy of drawings	The patient copies simple and complex drawings or shapes, such as squares, triangles, and more articulated objects.	Strengthens drawing accuracy and spatial perception.
		Assembly of virtual objects	Assemble parts of a complex object, such as a model or device, following step-by-step instructions.	Strengthens ability to follow assembly and construction sequences.	Cutting and gluing activities	The patient uses scissors, paper and glue to create shapes and compositions, practicing precise manipulation and hand control.	Improves hand–eye coordination and manual dexterity.
		Simulation of home environment	Rearrange the decor of a virtual room, choosing and placing furniture and decorations in a functional way.	Improves the ability to visualise and realise spatial configurations.	Block construction	The patient uses physical blocks to build towers or other structures, following a model or freely.	Enhances hand–eye coordination and spatial planning.
		Advanced dot connection	Complete figures by joining virtual dots in complex sequences.	Enhances the ability to follow and complete sequences.	Modeling activities	The patient uses plasticine to build three-dimensional models following detailed instructions, such as creating figures or buildings.	Enhances perception and spatial manipulation.
		Virtual Pathways	Follow and complete virtual paths that require coordinated movements through complex virtual environments.	Improves ability to orient and perform multiple tasks.	Drawing of maps	The patient creates or completes maps of simple environments, such as a room or neighborhood, by correctly placing key elements.	Strengthens the ability to organise and represent spatial configurations.

**Table 3:** Sociodemographic characteristics of the sample.

	All	EG	CG	*p*-Value
Participants	20	10 (50.0)	10 (50.0)	-
Male	10 (50.0)	4 (40.0)	6 (60.0)	0.65
Age (years)	48.30 ± 14.77	53.50 ± 8.87	43.10 ± 17.93	0.29
Education (years)	6.90 ± 4.83	11.10 ± 2.96	2.70 ± 1.16	**<0.001**

Legend: Experimental Group (EG); Control Group (CG). Continuous variables were expressed as mean ± standard deviation, whereas categorical variables are expressed as frequencies (percentages). Significant differences are in bold.

**Table 4:** Intra-and inter-group analysis between EG and CG.

		EG	CG	
		Median (1st Qu.–3rd Qu.)	Median (1st Qu.–3rd Qu.)	*p*-Value
MMSE	T0	16.00 (14.25–17.75)	22.00 (19.50–23.00)	**0.002**
	T1	20.50 (19.00–21.00)	23.50 (23.00–25.00)	**0.007**
	*p*-value	**0.002**	**0.002**	
FAB	T0	13.53 (12.63–14.00)	14.03 (12.25–14.50)	0.306
	T1	14.50 (13.63–16.50)	15.15 (13.63–13.00)	0.568
	*p*-value	**0.002**	**0.022**	
HRS-D	T0	12.00 (10.25–15.50)	12.00 (8.00–15.00)	0.761
	T1	11.50 (10.00–13.75)	12.00 (6.00–13.00)	0.704
	*p*-value	**0.012**	**0.021**	
Constructional Apraxia	T0	9.00 (8.25–9.75)	10.00 (6.75–12.00)	0.337
	T1	13.00 (12.00–13.75)	12.00 (10.25–13.00)	0.512
	*p*-value	**0.002**	**0.042**	
Ideomotor Apraxia	T0	6.50 (4.25–8.75)	12.00 (3.75–16.00)	0.363
	T1	14.50 (12.25–17.75)	18.50 (14.25–19.00)	0.383
	*p*-value	**0.002**	**0.009**	

Legend: Experimental Group (EG); Control Group (CG); Mini Mental State Examination (MMSE); Frontal Assessment Battery (FAB); Hamilton Rating Scale for Depression (HRS-D). Significant differences are in bold.

## Data Availability

The data that support the findings of this study are not openly available due to reasons of sensitivity and are available from the corresponding author upon reasonable request.

## References

[1] Zadikoff C., Lang A.E. (2005). Apraxia in movement disorders. Brain.

[2] Baumard J., Le Gall D. (2021). The challenge of apraxia: Toward an operational definition?. Cortex.

[3] Lane D., Tessari A., Ottoboni G., Marsden J. (2021). Body representation in people with apraxia post Stroke—An observational study. Brain Inj..

[4] Latarnik S., Stahl J., Vossel S., Grefkes C., Fink G.R., Weiss P.H. (2022). The impact of apraxia and neglect on early rehabilitation outcome after stroke. Neurol. Res. Pract..

[5] E Park J. (2017). Apraxia: Review and Update. J. Clin. Neurol..

[6] Bergqvist M., Möller M.C., Björklund M., Borg J., Palmcrantz S. (2023). The impact of visuospatial and executive function on activity performance and outcome after robotic or conventional gait training, long-term after stroke—As part of a randomized controlled trial. PLoS ONE.

[7] Li J., Yang L., Lv R., Kuang J., Zhou K., Xu M. (2023). Mediating effect of post-stroke depression between activities of daily living and health-related quality of life: Meta-analytic structural equation modeling. Qual. Life Res..

[8] Buxbaum L.J., Haaland K.Y., Hallett M., Wheaton L., Heilman K.M., Rodriguez A., Rothi L.J.G. (2008). Treatment of limb apraxia: Moving forward to improved action. Am. J. Phys. Med. Rehabil..

[9] Baak B., Bock O., Dovern A., Saliger J., Karbe H., Weiss P.H. (2015). Deficits of reach-to-grasp coordination following stroke: Comparison of instructed and natural movements. Neuropsychologia.

[10] Galeoto G., Polidori A.M., Spallone M., Mollica R., Berardi A., Vanacore N., Celletti C., Carlizza A., Camerota F. (2020). Evaluation of physiotherapy and speech therapy treatment in patients with apraxia: A systematic review and meta-analysis. La Clin. Ter..

[11] Satoh M., Mori C., Matsuda K., Ueda Y., Tabei K.-I., Kida H., Tomimoto H. (2017). Improved Necker Cube Drawing-Based Assessment Battery for Constructional Apraxia: The Mie Constructional Apraxia Scale (MCAS). Dement. Geriatr. Cogn. Disord. Extra.

[12] Patsaki I., Dimitriadi N., Despoti A., Tzoumi D., Leventakis N., Roussou G., Papathanasiou A., Nanas S., Karatzanos E. (2022). The effectiveness of immersive virtual reality in physical recovery of stroke patients: A systematic review. Front. Syst. Neurosci..

[13] Kim W.-S., Cho S., Ku J., Kim Y., Lee K., Hwang H.-J., Paik N.-J. (2020). Clinical Application of Virtual Reality for Upper Limb Motor Rehabilitation in Stroke: Review of Technologies and Clinical Evidence. J. Clin. Med..

[14] Ceradini M., Losanno E., Micera S., Bandini A., Orlandi S. (2024). Immersive VR for upper-extremity rehabilitation in patients with neurological disorders: A scoping review. J. Neuroeng. Rehabil..

[15] Wang L., Chen J.-L., Wong A.M., Liang K.-C., Tseng K.C. (2022). Game-Based Virtual Reality System for Upper Limb Rehabilitation After Stroke in a Clinical Environment: Systematic Review and Meta-Analysis. Games Health J..

[16] Cappadona I., Ielo A., La Fauci M., Tresoldi M., Settimo C., De Cola M.C., Muratore R., De Domenico C., Di Cara M., Corallo F. (2023). Feasibility and Effectiveness of Speech Intervention Implemented with a Virtual Reality System in Children with Developmental Language Disorders: A Pilot Randomized Control Trial. Children.

[17] De Luca R., Calderone A., Gangemi A., Rifici C., Bonanno M., Maggio M.G., Cappadona I., Veneziani I., Ielo A., Corallo F. (2024). Is Virtual Reality Orientation Therapy Useful to Optimize Cognitive and Behavioral Functioning Following Severe Acquired Brain Injury? An Exploratory Study. Brain Sci..

[18] De Luca R., Bonanno M., Rifici C., Pollicino P., Caminiti A., Morone G., Calabrò R.S. (2022). Does Non-Immersive Virtual Reality Improve Attention Processes in Severe Traumatic Brain Injury? Encouraging Data from a Pilot Study. Brain Sci..

[19] Barhorst-Cates E.M., Isaacs M.W., Buxbaum L.J., Wong A.L. (2022). Does spatial perspective in virtual reality affect imitation accuracy in stroke patients?. Front. Virtual Real..

[20] Rohrbach N., Krewer C., Löhnert L., Thierfelder A., Randerath J., Jahn K., Hermsdörfer J. (2021). Improvement of Apraxia with Augmented Reality: Influencing Pantomime of Tool Use via Holographic Cues. Front. Neurol..

[21] Folstein M.F., Folstein S.E., McHugh P.R. (1975). “Mini-Mental State”. A Practical Method for Grading the Cognitive State of Patients for the Clinician. J. Psychiatr. Res..

[22] Hamilton M. (1960). A rating scale for depression. J. Neurol. Neurosurg. Psychiatry.

[23] Appollonio I., Leone M., Isella V., Piamarta F., Consoli T., Villa M.L., Forapani E., Russo A., Nichelli P. (2005). The frontal assessment battery (FAB): Normative values in an Italian population sample. Neurol. Sci..

[24] Spinnler H., Tognoni G. (1987). Italian standardisation and calibration of neuropsychological tests (in Italian language). Ital. J. Neurol. Sci..

[25] De Renzi E., Faglioni P., Denes G., Pizzamiglio L. (1999). Apraxia. Handbook of Clinical and Experimental Neuropsy-Chology.

[26] Schmittmann V.D., Cramer A.O., Waldorp L.J., Epskamp S., Kievit R.A., Borsboom D. (2013). Deconstructing the construct: A network perspective on psychological phenomena. New Ideas Psychol..

[27] Morris M.C., Evans L.D., Rao U., Garber J. (2015). Executive function moderates the relation between coping and depressive symptoms. Anxiety Stress. Coping.

[28] Shi Y., Lenze E.J., Mohr D.C., Lee J.-M., Hu L., Metts C.L., Fong M.W., Wong A.W. (2024). Post-stroke Depressive Symptoms and Cognitive Performances: A Network Analysis. Arch. Phys. Med. Rehabil..

[29] Hoffman H.G., Doctor J.N., Patterson D.R., Carrougher G.J., Furness T.A. (2000). Virtual reality as an adjunctive pain control during burn wound care in adolescent patients. Pain.

[30] Howard M.C. (2017). A meta-analysis and systematic literature review of virtual reality rehabilitation programs. Comput. Hum. Behav..

[31] Sherrington C., Fairhall N.J., Wallbank G.K., Tiedemann A., A Michaleff Z., Howard K., Clemson L., Hopewell S., E Lamb S. (2019). Exercise for preventing falls in older people living in the community. Cochrane Database Syst. Rev..

[32] Laver K.E., Lange B., George S., Deutsch J.E., Saposnik G., Crotty M. (2017). Virtual Reality for Stroke Rehabilitation. Cochrane Database Syst. Rev..

[33] Maggio M.G., Stagnitti M.C., Rizzo E., Andaloro A., Manuli A., Bruschetta A., Naro A., Calabrò R.S. (2023). Limb apraxia in individuals with multiple sclerosis: Is there a role of semi-immersive virtual reality in treating the Cinderella of neuropsychology?. Mult. Scler. Relat. Disord..

[34] Rapaic D., Medenica V., Kozomora R., Ivanovic L. (2014). Limb apraxia in multiple sclerosis. Vojn. Pregl..

[35] Kamm C.P., Heldner M.R., Vanbellingen T., Mattle H.P., Müri R., Bohlhalter S. (2012). Limb Apraxia in Multiple Sclerosis: Prevalence and Impact on Manual Dexterity and Activities of Daily Living. Arch. Phys. Med. Rehabil..

[36] Goldenberg G., Daumüller M., Hagmann S. (2001). Assessment and therapy of complex activities of daily living in apraxia. Neuropsychol. Rehabil..

[37] Alashram A.R., Annino G., Aldajah S., Raju M., Padua E. (2022). Rehabilitation of limb apraxia in patients following stroke: A systematic review. Appl. Neuropsychol. Adult.

[38] Romano D., Tosi G., Gobbetto V., Pizzagalli P., Avesani R., Moro V., Maravita A. (2021). Back in control of intentional action: Improvement of ideomotor apraxia by mirror box treatment. Neuropsychologia.

[39] Kim J., Yi J., Song C.-H. (2017). Kinematic analysis of head, trunk, and pelvic motion during mirror therapy for stroke patients. J. Phys. Ther. Sci..

[40] Khrulev A., Kuryatnikova K., Belova A., Popova P., Khrulev S. (2022). Modern Rehabilitation Technologies of Patients with Motor Disorders at an Early Rehabilitation of Stroke (Review). Sovrem. Tekhnologii v Med..

[41] Pastore-Wapp M., Nyffeler T., Nef T., Bohlhalter S., Vanbellingen T. (2021). Non-invasive brain stimulation in limb praxis and apraxia: A scoping review in healthy subjects and patients with stroke. Cortex.

[42] Maggio M.G., Naro A., Manuli A., Maresca G., Balletta T., Latella D., De Luca R., Calabrò R.S. (2021). Effects of Robotic Neurorehabilitation on Body Representation in Individuals with Stroke: A Preliminary Study Focusing on an EEG-Based Approach. Brain Topogr..

[43] Calabrò R.S., Russo M., Naro A., De Luca R., Leo A., Tomasello P., Molonia F., Dattola V., Bramanti A., Bramanti P. (2017). Robotic gait training in multiple sclerosis rehabilitation: Can virtual reality make the difference? Findings from a randomized controlled trial. J. Neurol. Sci..

[44] Banz R., Bolliger M., Colombo G., Dietz V., Lünenburger L. (2008). Computerized Visual Feedback: An Adjunct to Robotic-Assisted Gait Training. Phys. Ther..

[45] Emedoli D., Arosio M., Tettamanti A., Iannaccone S. (2021). Virtual Reality Augmented Feedback Rehabilitation Associated to Action Observation Therapy in Buccofacial Apraxia: Case Report. Clin. Med. Insights Case Rep..

[46] De Luca R., Portaro S., Le Cause M., De Domenico C., Maggio M.G., Cristina Ferrera M., Giuffrè G., Bramanti A., Calabrò R.S. (2020). Cog-nitive rehabilitation using immersive virtual reality at young age: A case report on traumatic brain injury. Appl. Neuro-Psychol. Child.

[47] Park W., Kim J., Kim M. (2021). Efficacy of virtual reality therapy in ideomotor apraxia rehabilitation. Medicine.

[48] Qian Q., Zhao J., Zhang H., Yang J., Wang A., Zhang M. (2023). Object-based inhibition of return in three-dimensional space: From simple drawings to real objects. J. Vis..

[49] Stern Y. (2009). Cognitive reserve☆. Neuropsychologia.

